# Sodium Butyrate Ameliorates Streptozotocin-Induced Type 1 Diabetes in Mice by Inhibiting the HMGB1 Expression

**DOI:** 10.3389/fendo.2018.00630

**Published:** 2018-10-25

**Authors:** Yu Guo, Zheng Xiao, Yanan Wang, Weihua Yao, Shun Liao, Bo Yu, Jianqiang Zhang, Yanxiang Zhang, Bing Zheng, Boxu Ren, Quan Gong

**Affiliations:** ^1^Department of Immunology, School of Medicine, Yangtze University, Jingzhou, China; ^2^Clinical Molecular Immunology Center, School of Medicine, Yangtze University, Jingzhou, China

**Keywords:** sodium butyrate, HMGB1, Th1/Th2, type 1 diabetes, streptozotocin

## Abstract

Type 1 diabetes (T1D) is an autoimmune disease characterized by the immune cell-mediated progressive destruction of pancreatic β-cells. High-mobility group box 1 protein (HMGB1) has been recognized as a potential immune mediator to enhance the development of T1D. So we speculated that HMGB1 inhibitors could have anti-diabetic effect. Sodium butyrate is a short fatty acid derivative possessing anti-inflammatory activity by inhibiting HMGB1. In the current study, we evaluated the effects of sodium butyrate in streptozotocin (STZ)-induced T1D mice model. Diabetes was induced by multiple low-dose injections of STZ (40 mg/kg/day for 5 consecutive days), and then sodium butyrate (500 mg/kg/day) was administered by intraperitoneal injection for 7 consecutive days after STZ treatment. Blood glucose, incidence of diabetes, body weight, pancreatic histopathology, the amounts of CD4^+^T cell subsets, IL-1β level in serum and pancreatic expressions levels of HMGB1, and NF-κB p65 protein were analyzed. The results showed that sodium butyrate treatment decreased blood glucose and serum IL-1β, improved the islet morphology and decreased inflammatory cell infiltration, restored the unbalanced Th1/Th2 ratio, and down-regulated Th17 to normal level. In addition, sodium butyrate treatment can inhibit the pancreatic HMGB1 and NF-κB p65 protein expression. Therefore, we proposed that sodium butyrate should ameliorate STZ-induced T1D by down-regulating NF-κB mediated inflammatory signal pathway through inhibiting HMGB1.

## Introduction

Diabetes, as a non-communicable disease, has become the major cause of mortality and disease burden in the world. In recent decades, the incidence of diabetes has increased continuously that the estimated morbidity of diabetes in China was 10.9% in 2013 according to the latest published national survey (1). Type 1 diabetes (T1D) is characterized by the chronic hyperglycemia resulting from an immunologic disorder in which the autoreactive immune cells attack insulin-producing pancreatic β-cells (2). T1D is also known as juvenile-onset diabetes because it usually occurs in children and young adults (3). So far, the most suitable treatment for T1D is still insulin. However, the usage of insulin is restricted because of the inevitable chronical cardiovascular complications caused by unmanageable blood glucose and destruction of β-cells (4). Recently, alloislet transplantation has been becoming an appealing method for T1D treatment is, but therapeutic approaches are limited due to deficiency of donor (5). In recent onset of T1D, the insulitis is usually present that is characterized by immune cell inflammatory infiltration within pancreatic islets (6, 7), the process is thought to be important for autoimmune diabetes progression (8). Consequently, it is reasonable to prevent or treat T1D with some anti-inflammatory agents.

High-mobility group box 1 protein (HMGB1), a highly conserved chromosomal protein, can be passively released from damaged cells or secreted from immune cells. It was recognized as an innate signal to mediate the autoimmune initiation and progression of the systemic lupus erythematosus and rheumatoid arthritis (9, 10). HMGB1 was also recognized to be involved in the development of both type 1 and 2 diabetes (11–13). In the type 1 diabetes model, streptozotocin (STZ) is a widely used diabetogenic agent (14), Previous studies have shown that HMGB1 is activated and the expression is increased in STZ-induced diabetic mice (15, 16). In addition, Extracellular HMGB1 was also discovered as proinflammatory cytokine (17, 18). Comparing with early-acting role of TNF-α and IL-1, HMGB1 was identified as a late-acting cytokine to influence the progression of sepsis (19), so we speculated that anti-HMGB1 therapeutics would become an effective approach to treat inflammation-related autoimmune disease. For example, administration of anti-HMGB1 antibody reduced the diabetes incidence and delayed the onset of diabetes in NOD mice (11). Sodium butyrate, a short fatty acid derivative, is present in human diet such as butter and cheese and it is also notably produced in the large intestine through fermentation of dietary fiber (20). It can act as a direct HMGB1 antagonist, and showed the effects to attenuate myocardial ischemia/reperfusion injury (21), to protect against acute lung injury (ALI) induced by severe burn (22), and to reduce pancreas injury in severe acute pancreatitis (23), through modulating the expression of HMGB1.

Given the role of HMGB1 in T1D initiation and progression (11, 15, 16). And sodium butyrate, as a specific HMGB1 antagonist, has shown anti-inflammatory effect in various animal models, so whether sodium butyrate have some protective effect on the T1D development through inhibiting the HMGB1? In current study, we reported the potential beneficial effects of sodium butyrate in STZ-induced type 1 diabetes mouse model and its underlying molecular mechanisms.

## Materials and methods

### Animals

Male BALB/c mice (6–8 weeks, 25 ± 2 g) were purchased from Wuhan Centers for Disease Prevention & Control and the mice were bred and maintained in a pathogen-free facility, where kept the room temperature about 25°C, humidity about 50%, a standard 12 h dark/light cycles. The studies were in accordance with protocols approved by the Institutional Animal Care and Use Committee (IACUC) at the Yangtze University. The diabetes was induced by treating the male BALB/c mice multiple low doses of STZ (Sigma-Aldrich, Shanghai, China). Namely, the mice were received intraperitoneal injection of STZ at a dose of 40 mg/kg/day (dissolved in 0.1 mol/L citrate buffer, pH 4.5) for 5 consecutive days. Non-diabetic mice were received with an equal volume of vehicle. To observe the diabetic status of the mice, the non-fasting glucose from tail blood sampling were monitored by a glucose meter (OneTouch, LifeScan). Diabetes onset were diagnosed when blood glucose level >16.7 mmol (300 mg/dl) on 2 consecutive tests (24).

### Drug treatment

Sodium butyrate (Sigma-Aldrich, Shanghai, China) were dissolved in 0.9% sodium chloride solution and administered by intraperitoneal injection of 500 mg/kg/day at day 6 after STZ injection, the treatment were followed for 7 consecutive days.

### Serum collection

The mice were sacrificed at the end of experiment, after anesthetized with diethyl ether, the blood were collected using retro-orbital venous plexus puncture and then stayed at room temperature for 30 min, separated the serum through centrifugation at 12,000× g for 15 min at 4°C. The sera were kept at −70°C for ELISA.

### ELISA for cytokine assay

The amount of IL-1β in serum was determined using a commercial kit (MultiSciences, Hangzhou, China) according to the manufacturers' instructions.

### Histological and morphological analyses

The mice pancreases were removed and fixed in 4% formaldehyde at room temperature for 24 h, then the fixed tissues were infiltrated with paraffin, three series of 4 μm thick sections were prepared and subsequently subjected to standard hematoxylin and eosin staining to assess the pancreatic histopathologic changes.

### Flow cytometry analysis of CD4^+^T cell subsets

CD4^+^T cell subsets from the spleen and pancreatic lymph nodes (PLNs) were determined by flow cytometry. Briefly, the lymphocyte were isolated from fresh spleen and PLNs by mechanical dissociation, then centrifuged and adjusted the supernatant cell number to 2 × 10^6^, erythrocytes were lysed using red cell lysis buffer (Tiangen, Beijing, China), washed twice with RPMI-1640 (containing 10% FBS), added 0.5 μl PMA and BFA (MultiSciences, Hangzhou, China) and incubated for 5 h, followed by incubation with PE-labeled IL-4, PE-labeled IL-17A, FITC-labeled CD4, and APC-labeled IFN-γ (BD Pharmingen, Shanghai, China) at 4°C for 30 min. The cells were then subjected to flow cytometry analysis.

### Western blot analysis

Pancreas tissues were homogenized in the RIPA lysis buffer (MultiSciences, Hangzhou, China) containing various inhibitors. The lysates were separated by 10% SDS-PAGE and then electrotransferred onto polyvinylidene difluoride (PVDF) membranes. The membranes were incubated with primary antibody for the protein of interest or anti-β-Actin, the membranes were washed with Tris-buffered saline with Tween and incubated with HRP-conjugated secondary antibody. Immunoreactivity was detected using an enhanced chemiluminescence reagent (MultiSciences, Hangzhou, China).

### Statistical analysis

Results are shown as mean ± standard deviation (SD). Graphical presentation and statistical analyses were carried out with GraphPad Prism software. The Student's *t*-test were used for comparison of the mean for two groups (plasma glucose and body weight). The difference of diabetes onset between groups were determined using the log-rank (Mantel–Cox) test. Comparisons between groups for cytokine secretion, the amount of CD4^+^T cell subsets and western blot were performed by one-way ANOVA. *P* < 0.05 was considered statistically significant.

## Results

### Administration of sodium butyrate decreases plasma glucose and delays the onset of diabetes

**Figure 1:**
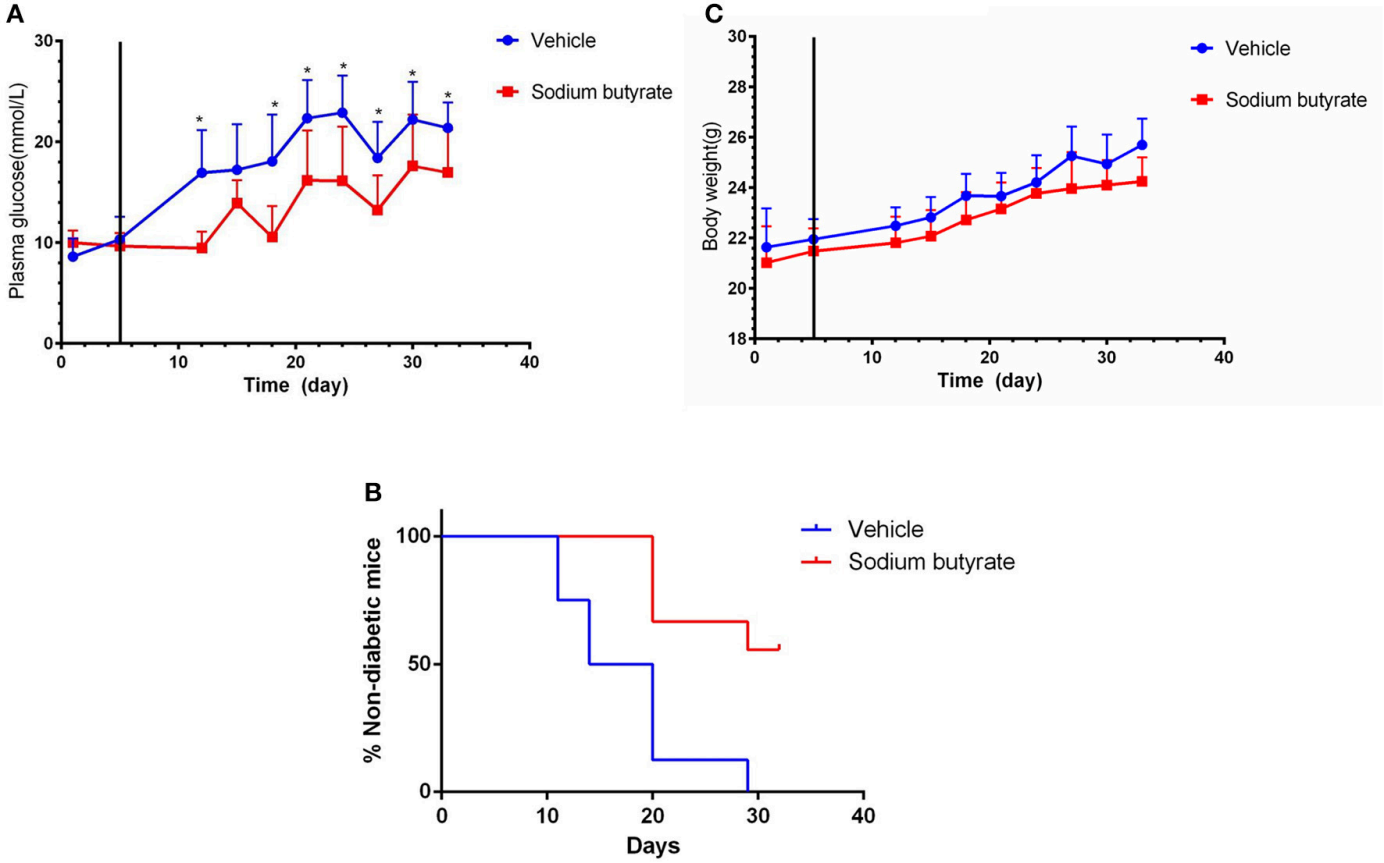
Sodium butyrate treatment decreases blood glucose level and delays the onset of diabetes. Male BALB/c mice received STZ treatment by i.p. route at a dose of 40 mg/kg/day for 5 consecutive days. Sodium butyrate (dose of 500 mg/kg/day, *n* = 9) or vehicle treatment (*n* = 8) from day 6 (solid line labeled) for 7 consecutive days after the last STZ injection. **(A)** STZ induced diabetic mice exhibited lower blood glucose when receiving sodium butyrate treatment compared with vehicle from day 11 (*p < 0.05). **(B)** Treatment with sodium butyrate decreased diabetes incidence and delayed the onset of diabetes (*p < 0.01). **(C)** Sodium butyrate treatment had no effect on the body weight of the diabetic mice.

To determine the effect of sodium butyrate on diabetes, the mice were intraperitoneal injected with 500 mg/kg sodium butyrate after the STZ injection. Vehicle-treated mice developed hyperglycaemia within 7 day after the last STZ injection, whereas the mice administrated with sodium butyrate exhibited lower non-fasting serum glucose levels compared with vehicle group (Figure 1A). Although sodium butyrate can't block the progression of diabetes, sodium butyrate treatment significantly postponed the development of diabetes (Figure 1B). The incidence of diabetes (non-fasting blood glucose level > 16.7 mmol) was first observed in vehicle group at day 12 compared with sodium butyrate group at day 21. In addition, we have also monitored the effect of sodium butyrate on the body weight and food intake of diabetic mice, but there was no significant difference between vehicle and sodium butyrate group (Figure 1C and Supplementary Figure 1).

### Pancreatic histopathologic changes are improved by sodium butyrate

**Figure 2:**
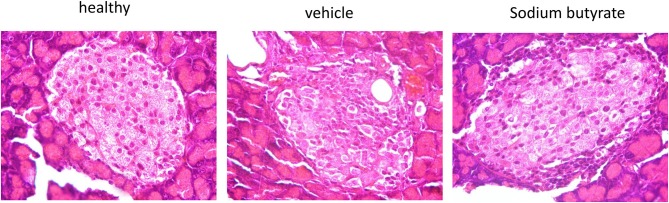
Sodium butyrate treatment ameliorates the pancreatic histopathologic changes. Hematoxylin and eosin staining of pancreases from healthy mice, vehicle group, and sodium butyrate treated mice (H&E staining, magnification 1000×).

Histological examination of mice pancreases were performed to evaluate the effect of sodium butyrate on STZ-induced mice. As shown in Figure 2, the healthy mice had intact islet morphology. However, islet boundary became a little vague and cell number inside islet decreased in diabetic mice. Moreover, heavy inflammatory cell infiltration at one side was evident. Here, although sodium butyrate treatment could not prevent the inflammatory cells infiltration, morphology of islet was improved when compared with vehicle group.

### The ratio of Th1/Th2 and Th17 cells are regulated by sodium butyrate

**Figure 3:**
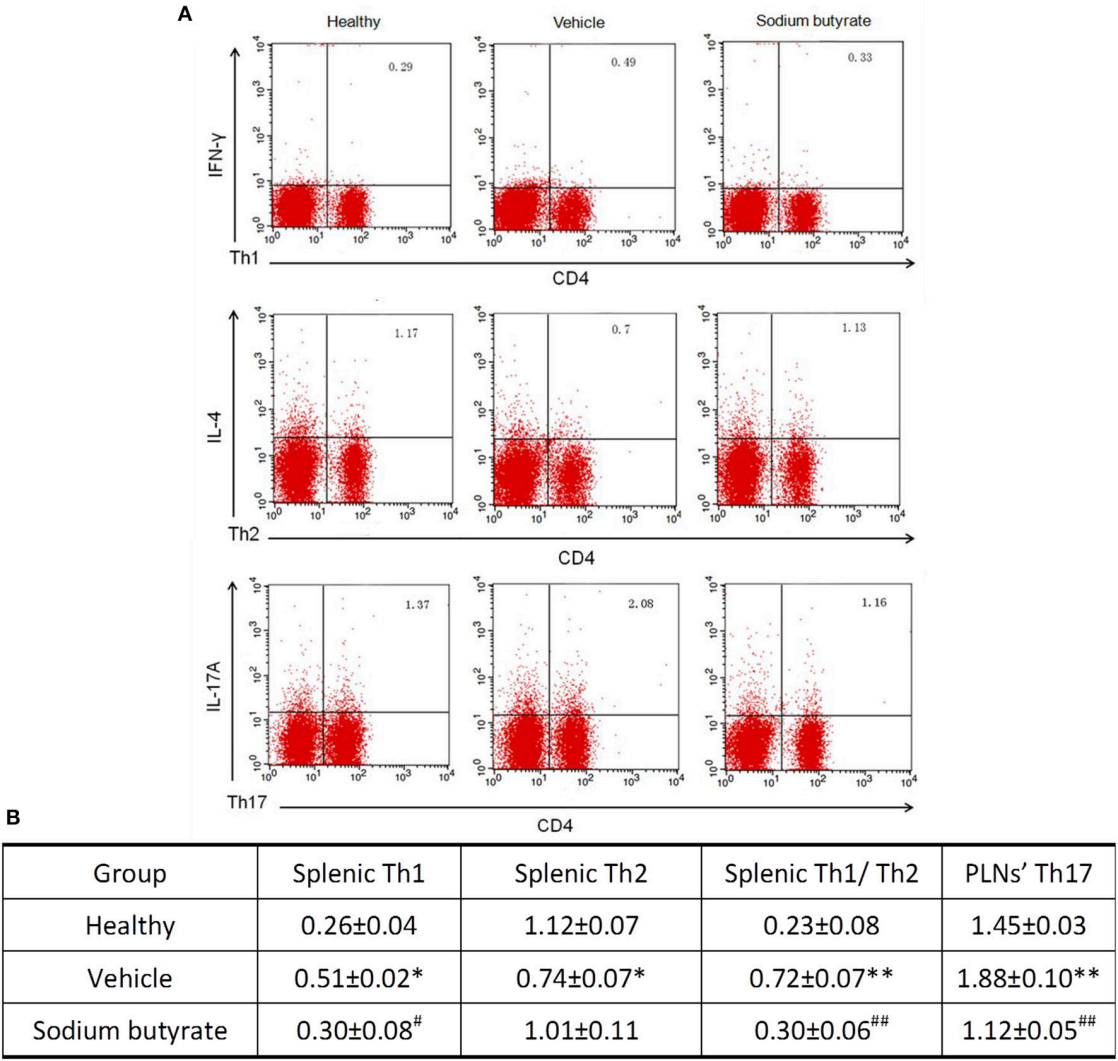
Sodium butyrate treatment regulates CD4^+^ T cell subsets **(A)**. Splenic and PLNs' lymphocyte originated from sodium butyrate treated mice (*n* = 5), vehicle group (*n* = 5) and healthy mice (*n* = 3) were harvested for CD4, IL-4, IFN-γ, and IL-17A staining. The percentages of Th1, Th2, and Th17 cells were determined by flow cytometric analysis **(B)**. The data are listed in the table as means ± SD of three independent experiment. The ratio of splenic Th1/Th2 and Th17 cell in PLNs was significantly higher than healthy mice, whereas sodium butyrate treatment restored unbalanced Th1/Th2 and regulated Th17 cells to normal level. *p < 0.05, **p < 0.01 vs. healthy group, ^#^*p* < 0.05, ^##^*p* < 0.01 vs. vehicle group.

It has been reported that the pathogenesis of some inflammatory diseases are associated with the imbalance of Th1/Th2 and Th17 cells (25–27). We determined whether the anti-diabetic effect of sodium butyrate are related with the changes of Th1/Th2 in the spleen and Th17 cells in PLNs. As shown in Figure 3, vehicle treated diabetic mice showed a higher percentage of Th1 and lower percentage of Th2 compared with non-diabetic mice. More importantly, the ratio of Th1/Th2 and Th17 cells in diabetic mice are higher than non-diabetic mice. Whereas, sodium butyrate regulated the increased ratio to a relative low level that was closed to health non-diabetic mice. These results demonstrated that sodium butyrate should exhibit the anti-diabetic effect through modulating the unbalanced Th1/Th2 and decreased Th17 cells to the normal level.

### Proinflammatory cytokine IL-1β are down-regulated in sodium butyrate treated diabetic mice

**Figure 4:**
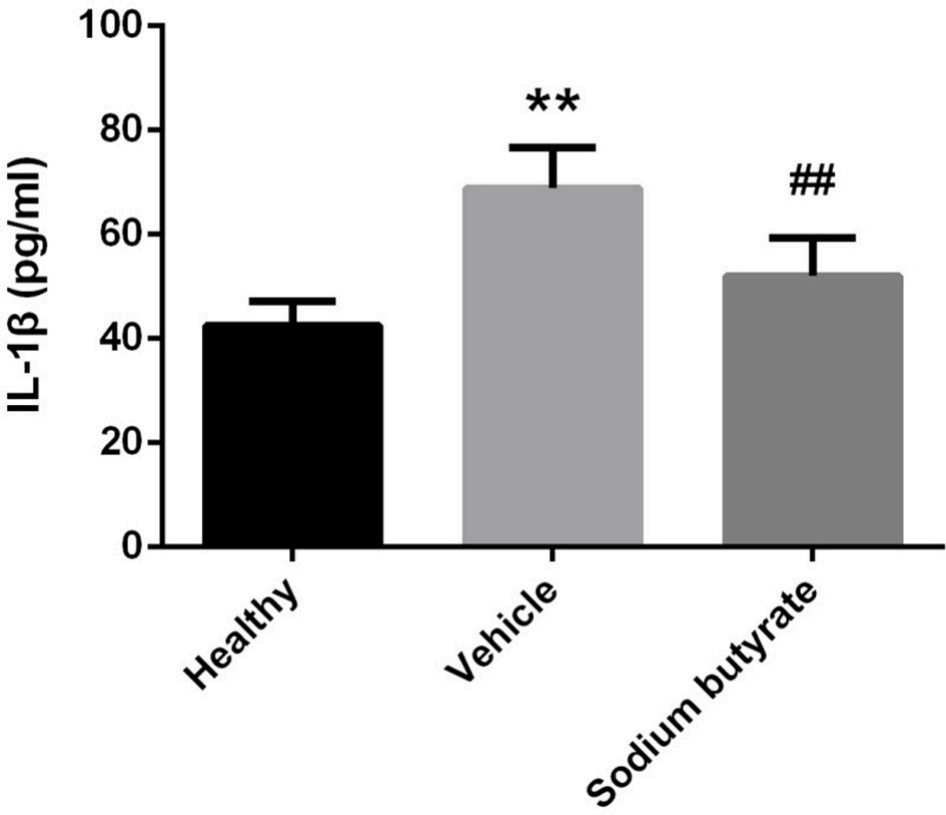
Sodium butyrate down-regulates proinflammatory cytokine IL-1β expression in diabetic mice. Levels of serum cytokine IL-1β in sodium butyrate treatment (*n* = 9), vehicle group (*n* = 8) and healthy group (*n* = 5) mice were assessed by ELISA. The IL-1β level is increased in diabetic mice compared with normal, whereas sodium butyrate inhibit its expression. **p < 0.01 vs. healthy group, ^##^*p* < 0.01 vs. vehicle group.

Considering the role of proinflammatory cytokines on the pathogenesis of diabetes, population with detectable levels of circulating IL-1β cytokines have increased risk to develop diabetes (28–30). We speculate that sodium butyrate could relieve diabetes reflected by decreased level of IL-1β. As expected, diabetic mice showed increased levels of IL-1β, but sodium butyrate treatment significant decreased the level (Figure 4). The data suggested that sodium butyrate could inhibit proinflammatory response of diabetes.

### Sodium butyrate inhibits the pancreatic HMGB1 and NF-κB p65 protein expression

**Figure 5:**
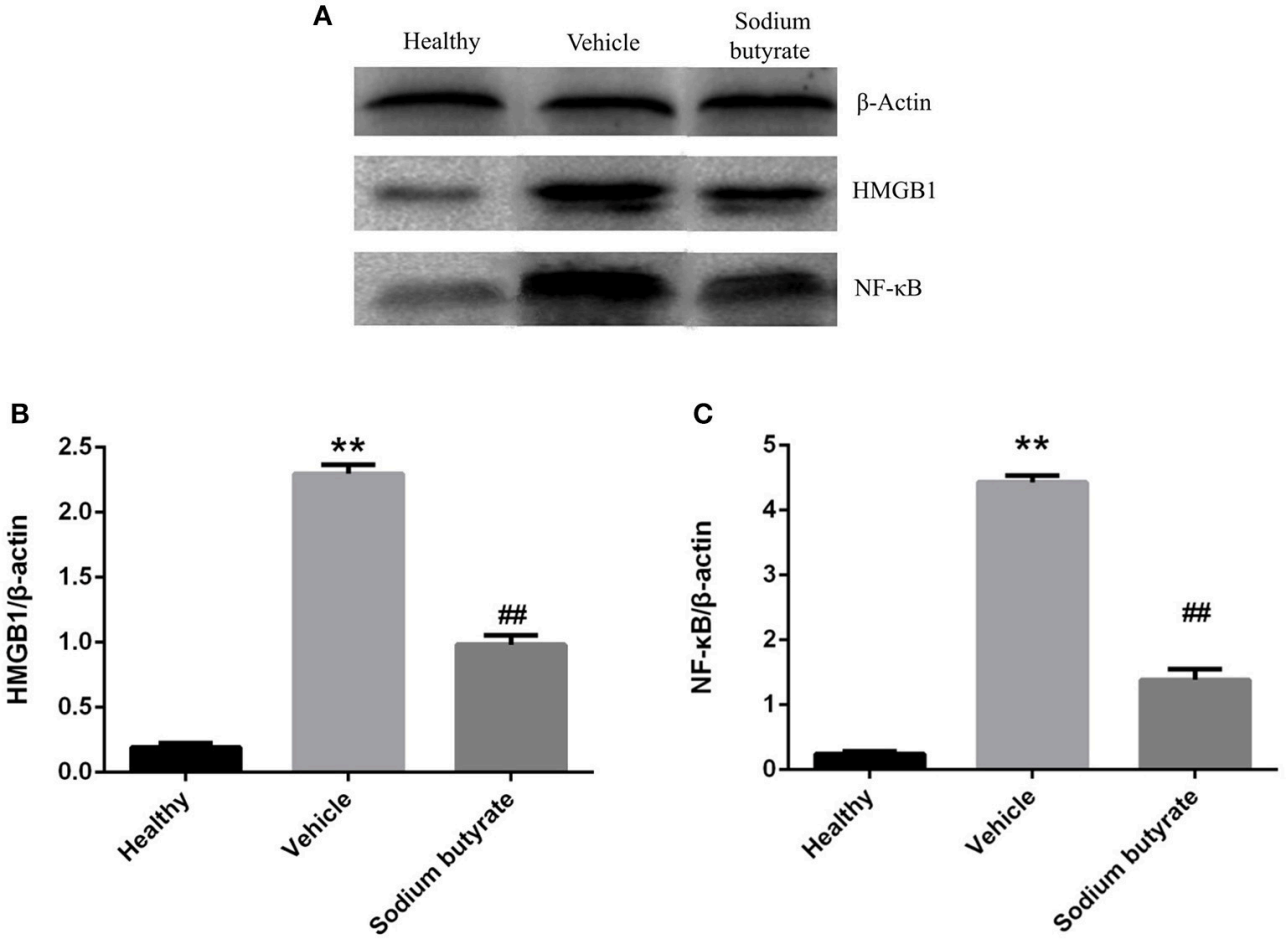
Sodium butyrate inhibits the pancreatic HMGB1 and NF-κB p65 protein expression **(A)**. Western blot analysis of pancreatic HMGB1 and NF-κB p65 protein expression from sodium butyrate group (*n* = 5), vehicle group (*n* = 5), and healthy group mice (*n* = 3). Both HMGB1 and NF-κB p65 expression were increased in diabetic mice compared with healthy group, however, sodium butyrate down-regulated their expression **(B,C)**. Quantitative analysis of A results are representative of three independent experiments and data represent the mean ± SD. **p < 0.01 vs. healthy group, ^##^*p* < 0.01 vs. vehicle group.

Pancreatic HMGB1 and NF-κB p65 protein expression were analyzed by western blot. Results showed that both HMGB1 and NF-κB p65 protein expression were up-regulated in diabetic mice compared with healthy non-diabetic mice, in contrast, the protein expression was markedly down-regulated in the mice treated with sodium butyrate compared with diabetic mice (Figure 5).

## Discussion

Current strategies for T1D treatment include lifelong insulin delivery, maintaining normal glycemic level, eating healthy foods, and keeping to a healthy weight. As is known to us, people with diabetes have a higher risk to develop one or more complications, so it is important to control and maintain the blood glucose within the normal level.

HMGB1, a highly conserved non-histone nuclear protein, was proven to be involved in the pathogenesis of inflammatory and autoimmune disease (31), and it can serve as endogenous alarmin to alert the innate immune system to promote host defense or tissue repair. The role of HMGB1 in autoimmune disease was first confirmed in rheumatoid arthritis (RA) (32). Extranuclear HMGB1expression was increased in the synovia of patients and animal models with rheumatoid arthritis (RA), and blockade of HMGB1 expression in experimental animal models can attenuate the RA (32, 33). In addition, HMGB1 was also involved in pathogenesis of systemic lupus erythermatosus (SLE), the patients with SLE show increased level of HMGB1 in the epidermis, and the increased plasma levels of HMGB1 correlated closely with disease activity (34). T1D is also an autoimmune disease characterized by destruction of the insulin secreting β-cells, previous study has shown that HMGB1 seems to be involved in T1D pathogenesis and it can act as a potent innate alarmin to mediate the initiation and progression during T1D development in NOD mice, a model of spontaneous T1D (11). When NOD mice were treated with HMGB1 neutralizing antibody, the insulitis progression was significantly inhibited and the diabetes incidence was also decreased (11). HMGB1 was also thought to be involved in the pathogenesis of type 2 diabetes (T2D) (35). It has been reported that serum HMGB1 level was increased in patients with T2D, and *in vitro* study showed that high glucose can activate HMGB1 expression in mesangial cells (35). A recent investigation about the effect of HMGB1 on high concentration glucose induced mesothelial cells (MCs) injury also demonstrated that high glucose promoted HMGB1 translocation and secretion from the nucleus of MCs (36). Moreover, it has also been reported that high glucose can induce retinal pericytes, vascular smooth muscle cells and human aortic endothelial cells to secret HMGB1 (37–39). So it is undoubted that HMGB1 expression could be activated under high glucose induction.

Based on the potential role of HMGB1 in the pathogenesis of diabetes, It was presumed that HMGB1 inhibitors would probably affect the diabetes onset. Sodium butyrate is a well-known short fatty acid derivative and exhibits good anti-inflammatory property through inhibiting HMGB1 expression. It has been proven that sodium butyrate showed protective effect in myocardial ischemia/reperfusion, severe sepsis and ALI by inhibiting HMGB1 (21, 22, 40, 41). Sodium butyrate can also improve the performance of diabetic complications (42, 43). For example, sodium butyrate showed protective effect against diabetic nephropathy (DN) (42). In a high fat diet (HFD)-induced type II diabetic model, cardiac function and metabolic dysfunction were improved by sodium butyrate (43). In addition, sodium butyrate can prevent the insulin resistance in HFD-induced obese mice (44).

CD4^+^ T helper (Th) cells are major T cell subsets that play a vital role in mediating immune responses. According to cytokine production and specialized functions, the Th cells can be classified into at least four distinct Th phenotypes (Th1, Th2, Th17, and T-regulatory cells). Th1 cells were responded for cellular immunity through secreting interferon (IFN)-γ (45), Th1 cells were thought to be involved in the insulin-producing β-cell destruction in the pancreatic islets (46). Whereas, Th2 cells were mainly involved in mediating humoral immunity and would actually protect against autoimmune disease (47). Induction of Th2 cells could lead to dominant protective effect against T1D development (48). Previous study had shown that Th1/Th2 imbalance could contribute to pathogenesis of some autoimmune diseases (26). Th17 cells, another subset of CD4^+^T cells, can produce proinflammatory cytokine IL-17 and induce inflammation to mediate autoimmune pathology. Some evidence indicated that Th17 cell and its related cytokines had significant effects on the onset and progression of T1D in both human and animals (25, 27).

**Figure 6:**
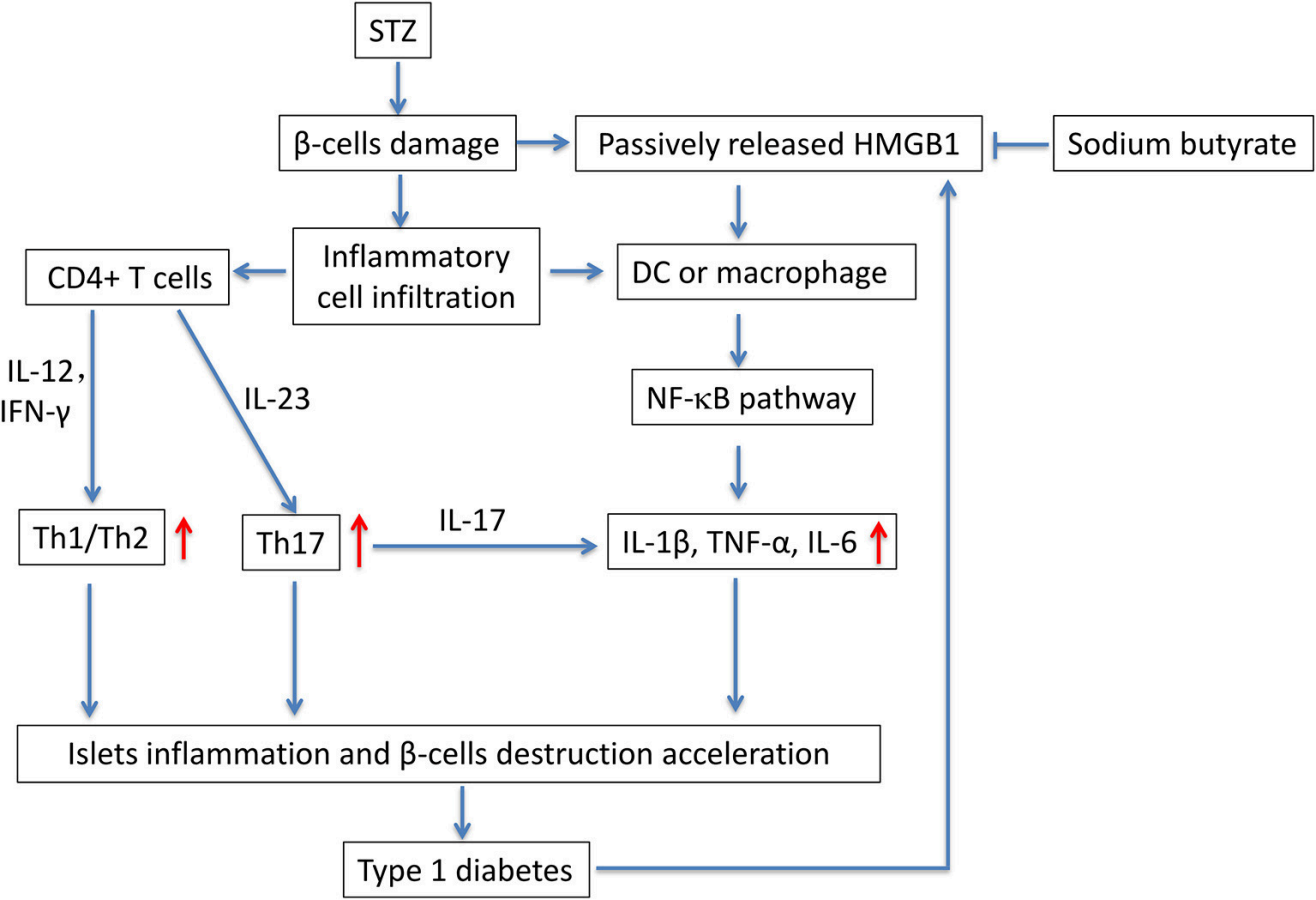
The potential mechanism of sodium butyrate on STZ-induced type 1 diabetes. STZ, specifically destroys pancreatic β-cells. The damaged β-cells passively release HMGB1 (11). At the same time, the islets are infiltrated by inflammatory cells such as DCs, macrophages and T cells. Naïve CD4^+^ T cells are differentiated into effector Th1 and/or Th17 cells based on their cytokine microenvironment. In addition, the released extracellular HMGB1 targets DCs or macrophage via the corresponding surface receptor(s), induces a signaling cascade and activates NF-κB pathway (12), therefore leading to the production of proinflammatory cytokines IL-1β, TNF-α, and IL-6 (49). the unbalanced Th1/Th2/Th17 and proinflammatory cytokines further accelerate the islets inflammation and β-cells destruction and lead to the onset of type 1 diabetes. Sodium butyrate, as a direct HMGB1 antagonist, could down-regulate the expression of HMGB1 and mediate the balance of Th1/Th2/Th17 paradigm, thus attenuating type 1 diabetes.

In this study, we evaluated the potential anti-diabetic effect of sodium butyrate in a STZ-induced T1D mice model. Multiple low dose injection of STZ led to pronounced pancreatic insulititis, followed by β-cell destruction and plasma glucose elevation (14), then the HMGB1 was passively released from damaged β-cell (11), and it acted as proinflammatory cytokine to enhance the inflammatory response. We found that sodium butyrate exhibited the protective effect on streptozotocin-induced type 1 diabetes in mice. Sodium butyrate treatment decreased the level of plasma glucose and delayed the onset of diabetes. In order to investigate the mechanism of beneficial effects of sodium butyrate for T1D, we first analyzed the phenotypes of CD4^+^ T cells. The results showed that ratio of Th1/Th2 and Th17 were increased in diabetic mice, and sodium butyrate treatment significantly decreased the proportion of Th1/Th2 in spleen and Th17 from PLNs. As aforementioned, Th1 and Th17 cell are closely associated with onset and development of T1D. So it is not difficult to speculate that sodium butyrate could recover the balance of Th1/Th2 and inhibit theTh17 cell to the normal level. Th17 initiated the inflammation through stimulating the production of proinflammatory cytokines, IL-1β, IL-6, and TNF-α and in turn accelerated β-cell destruction (49). And our results demonstrated that sodium butyrate treatment did decrease the level of IL-1β in serum. To further address the underlying mechanism of sodium butyrate, we determined the NF-κB p65 expression. Because NF-κB is an important transcription factor that regulates the inflammatory gene expression. An *in vitro* study showed high glucose mimicking diabetes can lead to the activation of NF-κB and subsequent increased expression of inflammatory chemokines and cytokines (35, 50). In our study, NF-κB p65 expression was increased in diabetic mice, indicating NF-κB signaling pathway is involved in pathogenesis of the streptozotocin-induced type 1 diabetes model. Whereas treatment with sodium butyrate can inhibit the NF-κB p65 levels. Therefore, the main results can be summarized in Figure 6. Briefly, STZ directly targets and destroys pancreatic β-cells, then HMGB1 was passively released from damaged β-cells (11), and interacts with DCs or macrophage via the corresponding surface receptor(s), induces a signaling cascade and activates NF-κB pathway (12), therefore leading to the production of proinflammatory cytokines (such as IL-1β), the cytokines together with unbalanced Th1/Th2/Th17 accelerate the islets inflammation and β-cells destruction and finally develop into diabetes, high glucose would promote HMGB1 expression and further aggravate the diabetic condition through positive feedback effect of HMGB1 and high glucose. Whereas, sodium butyrate, as a direct HMGB1 antagonist, could down-regulate the expression of HMGB1 and mediate the balance of Th1/Th2/Th17 paradigm, thus attenuating type 1 diabetes.

Additionally, emerging evidences have implicated that gut bacterial composition may be associated with disease development and progression of T1D in both animal and human (51, 52). And a recently study has also suggested that short chain fatty acids (including sodium butyrate) treatment to rat breeders can ameliorates T1D in the offspring through reshaping the intestinal microbiota (53). So we speculate that sodium butyrate, to some extent, could restore the balance of intestinal flora to maintain metabolic homeostasis in the STZ-induced T1D mice. And we would perform experiments to observe the effect of sodium butyrate on intestinal microbiota composition in the future, such as *Lactobacillus* and *Bifidobacterium* that are associated with the progression of T1D (54).

Altogether, our data suggest that sodium butyrate ameliorates STZ-induced type 1 diabetes. The beneficial effects could be attributed to the effects of sodium butyrate on restoring the unbalanced Th1/Th2/Th17 paradigm and inhibiting NF-κB-mediated inflammatory pathway. Therefore, sodium butyrate would become a beneficial dietary supplementation for T1D patients.

## Author contributions

YG and ZX searched related literatures and made the mice model of STZ-induced T1D. WY was responsible for blood glucose monitoring. SL and BY performed the ELISA and western blot. YW and JZ performed the flow cytometry analysis and statistical analysis. YZ was responsible for histological and morphological analysis. QG and BR designed the experiment and supervised the project. BZ wrote the manuscript with contribution from all authors.

### Conflict of interest statement

The authors declare that the research was conducted in the absence of any commercial or financial relationships that could be construed as a potential conflict of interest.
