# Supplementary figures and images for: Sodium Butyrate Ameliorates Streptozotocin-Induced Type 1 Diabetes in Mice by Inhibiting the HMGB1 Expression
Source: Front Endocrinol (Lausanne). 2018 Oct 25;9:630. doi: 10.3389/fendo.2018.00630 (PMC6209660; doi:10.3389/fendo.2018.00630)

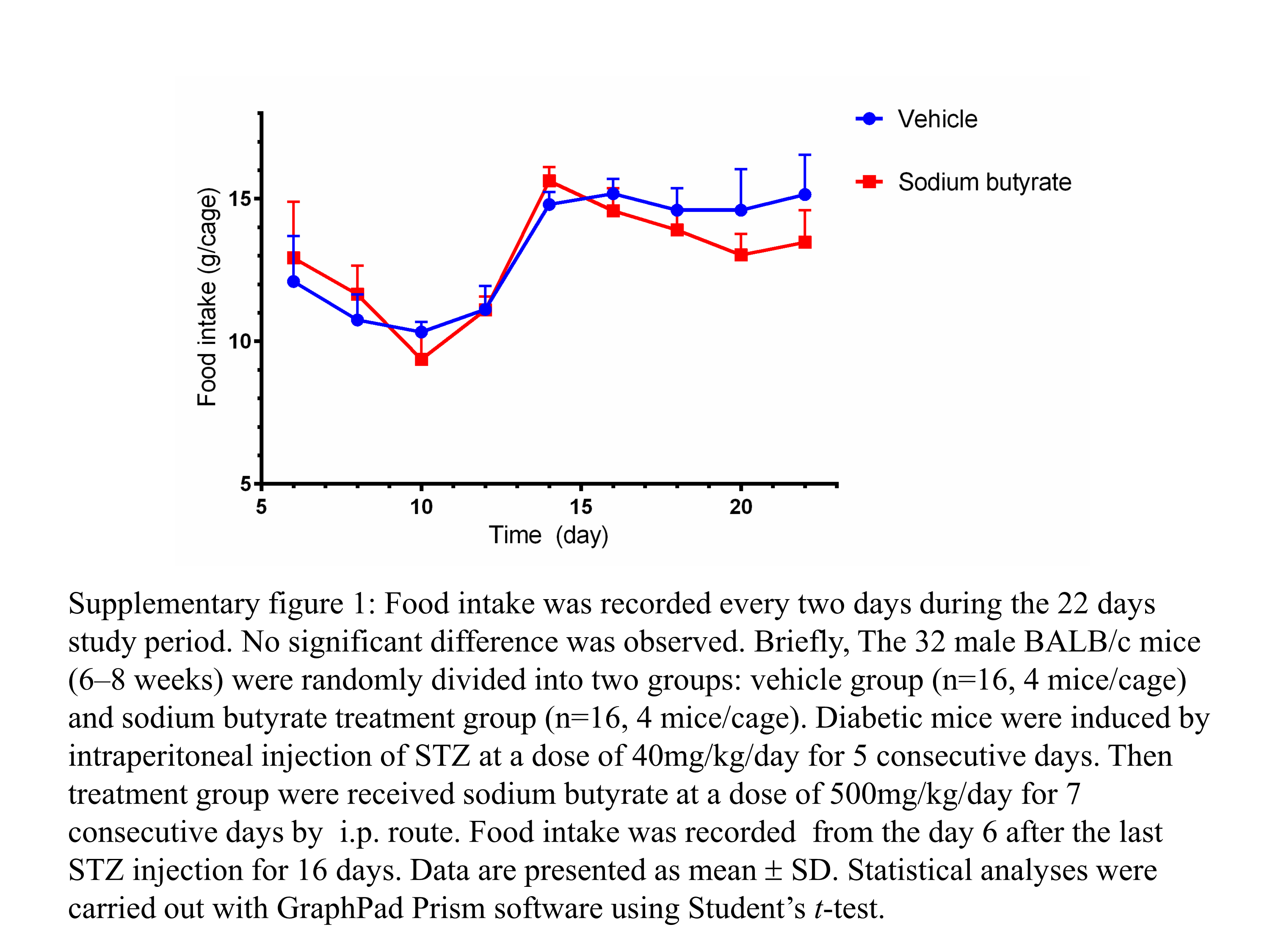

Supplement: Supplementary file 1 [file Image_1.TIF]
